# Supplementary material for: Using mechanistic models and machine learning to design single-color multiplexed nascent chain tracking experiments
Source: Front Cell Dev Biol. 2023 May 30;11:1151318. doi: 10.3389/fcell.2023.1151318 (PMC10267835; doi:10.3389/fcell.2023.1151318)
Supplement: Supplementary file 1 [file DataSheet1.PDF]

Supplementary Material

1 SUPPLEMENTARY DATA

1.1 Photobleaching and tracking experiment

Classification is possible with photobleaching and tracking errors

To examine the effects of photobleaching on classification, simulated videos were subjected to 11 different photobleaching rates before processing. 75 Simulated cell videos of 350 frames at 1 second frame interval containing 25 spots of KDM5B and 25 spots of P300 with k_i = 0.06 s^{-1} and k_e = 5.33 $aa \cdot s^{-1}$ were simulated for each photobleaching rate ranging from 0.01% lost per 5 seconds to 5% lost per 5 seconds. Diffraction limited RNA spots were added to the channel zero (red) for simulation of particles for tracking; These RNA tag spots were colocalized with the channel one (green) spots for their corresponding NCT protein signal. Photobleaching was added post simulation by multiplying the resultant videos by the normalized photobleaching curves shown in Supplemental Figure S4B. Sample videos are shown in Supplemental Figure S4A for each photobleaching rate. Extrema were removed from each video channel by normalizing intensities to the 0.05th and 99.9th percentile of intensity. Particle tracking was performed on each video after image processing. The video's red channel was filtered for spots by applying a bandpass filter (skimage.difference_of_gaussians, low_sigma = 0.1, high_sigma = 5, truncate = 3) and Laplace of Gaussian's filter (scipy.ndimage.gaussian_laplace, sigma = 1.5). The filtered video was passed to Trackpy (**?**). Trackpy was iteratively called on the filtered video's red channel with increasing until the number of spots detected levels off (approximated derivative of # spots detected / intensity threshold = 0). The location where the number of spots detected levels off is used as the final intensity threshold. Tracking efficiency results are shown in Supplemental Figure S3.C. This iterative Trackpy approach on average finds 120% of the true simulated number of spots – consisting of false positive spots $\sim$33%, real spots and real spots whose trajectories are not correctly linked resulting in multiple particle tracks from one spot, $\sim$66%. To filter out false positive spots and short detected trajectories, all spots were then matched with their minimum squared error divided by the length of the tracked trajectory (time normalized squared error) to the true locations from their simulated video. Spots with an error lower than 3 pixels were considered "real" particles and kept for classification tests, $\sim$80% of real spots were recovered in some form with this error matching. Additionally, any spot not detected for $\geq$300 seconds was excluded from classification. Percentage of spots detected and kept for classification as a function of photobleaching rate is shown in the purple line in Supplemental Figure S4C. Tracking is around 40% efficient at recovering long trajectory true spots unless the videos significantly photobleach before the threshold of 300 seconds. All final spots used for classification were down-sampled to 60 frames at a 5 second frame interval for classification. Classification was performed on the "perfect tracking" using 2000 training spots / 500 withheld spots. The "realistic tracking" data set was trained on the maximum possible recovered spots from the 75 total cells while withholding 500 spots for validation. Supplemental Figure S4D shows the accuracy versus photobleaching rates with "perfect tracking" and with the "realistic tracking" from the Trackpy pipeline. Test accuracy stays level across photobleaching rates for both types of tracking; This would change with longer videos that can supplement their classification with the frequency information of the autocorrelation – the videos would be easier to classify with lower photobleaching rates where a better autocorrelation can be measured. However, at only 300 seconds of video, there is not enough time to acquire adequate dwell time information and the classifier is exclusively using intensity differences to tell spots apart; With a consistent

photobleaching across genes, these intensity differences are also consistent at any given time point (until all intensity is lost). Greater in-depth simulations of photobleaching such as differing photobleaching rates for different elements of the simulation or other photobleaching models and their effect on classification can be explored in the future.

1.2 Figures

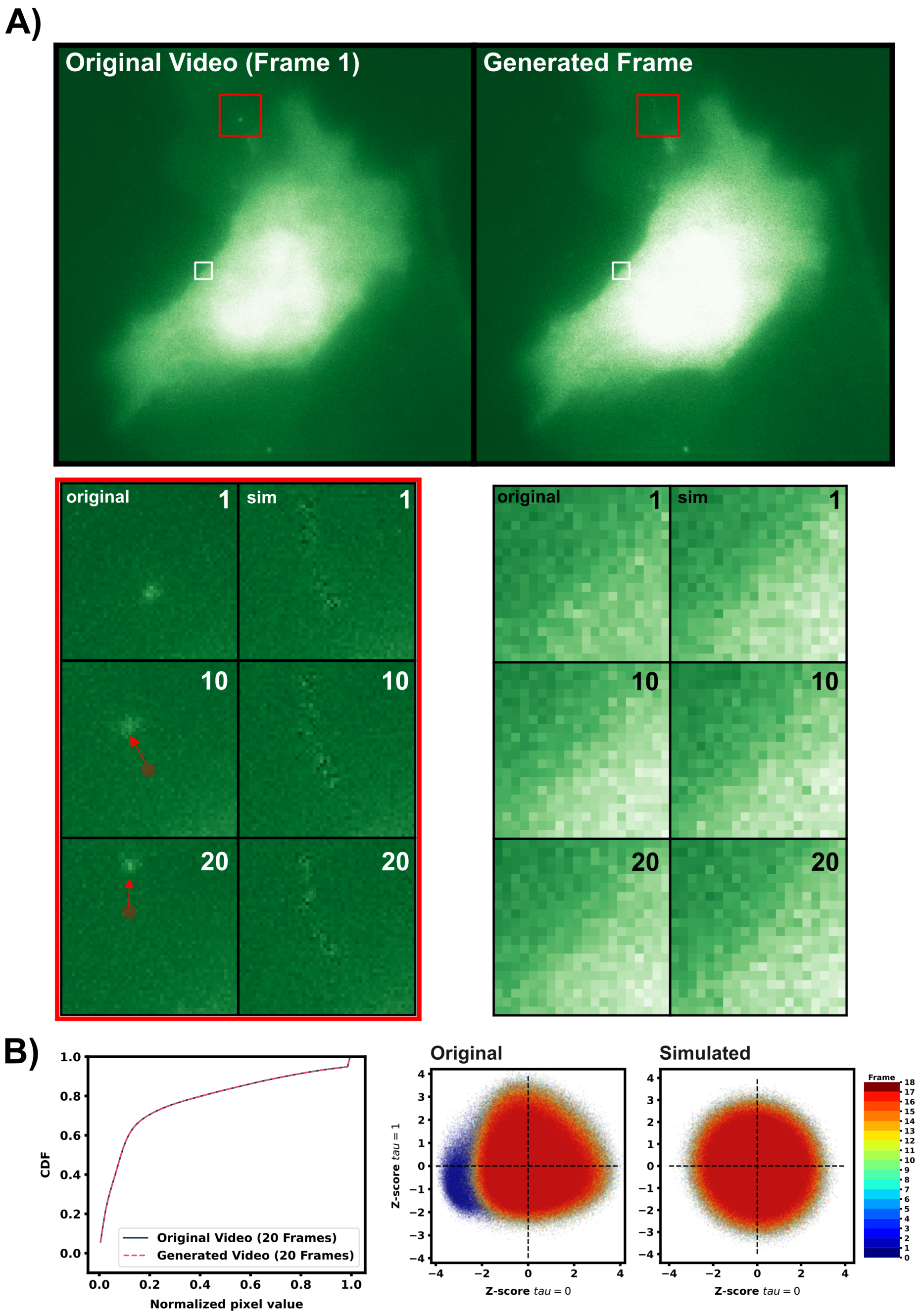

Figure S1. Gaussian generation of simulated cell backgrounds. Each pixel within the original blank cell video (20 frames) is fit to its own Gaussian distribution. New frames are generated pixel by pixel by pulling new values from that array of Gaussians. Standard deviations above the 95th quantile of the total video's per pixel standard deviation are set to that 95th quantile value – that way problem pixels that may be dark for 19 out of 20 frames do not result in a massively wide Gaussian to pull from. For the purposes of visualization, videos were min max normalized by the 95th quantile intensity and the 1st quantile value of the original video. The red square highlights a region where a large moving feature in the original video is lost by the Gaussian generation approach. Large bright features that move over the course of 20 frames are represented as a larger standard deviation and a slightly higher Gaussian mean, resulting in a smeared "noisy" region in the generated videos that overlays the course of the original bright feature. The white box shows an example highlighted area with no moving feature. Below shows the CDF of each video's histogram of normalized pixel values. We note that for all 7 background videos the first frame is noticeably dimmer than all the rest (blue region in panel B middle).

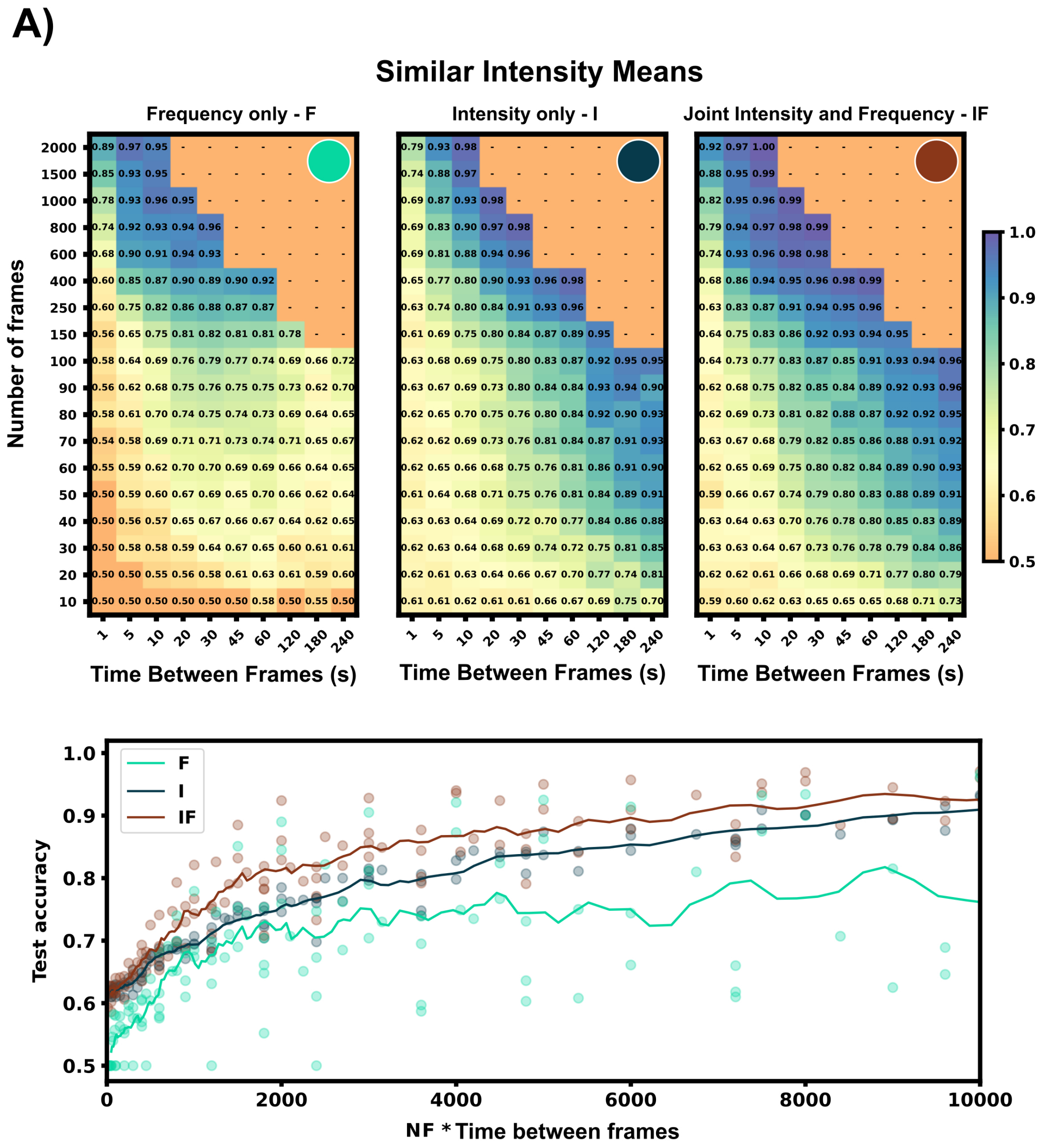

Figure S2. (Top) Accuracy of separate architecture halves (I, F) and joint architecture (IF) across a large range of frame rates and number of frames. Heat maps show each architecture half or the joint architecture applied on a NCT data set with different frequency content (decorrelation times, τ= 354s vs τ=517s) and similar intensity means (4.7 UMP vs 6 UMP). (Bottom) Using both architecture halves increases average test accuracy across all conditions vs the individual feature architectures. All test accuracies from the heat maps above are plotted vs their video length (NF * Time between frames) for the full architecture (IF), and each half architecture (I, F). A trend-line was generated with a moving average of 100 seconds.

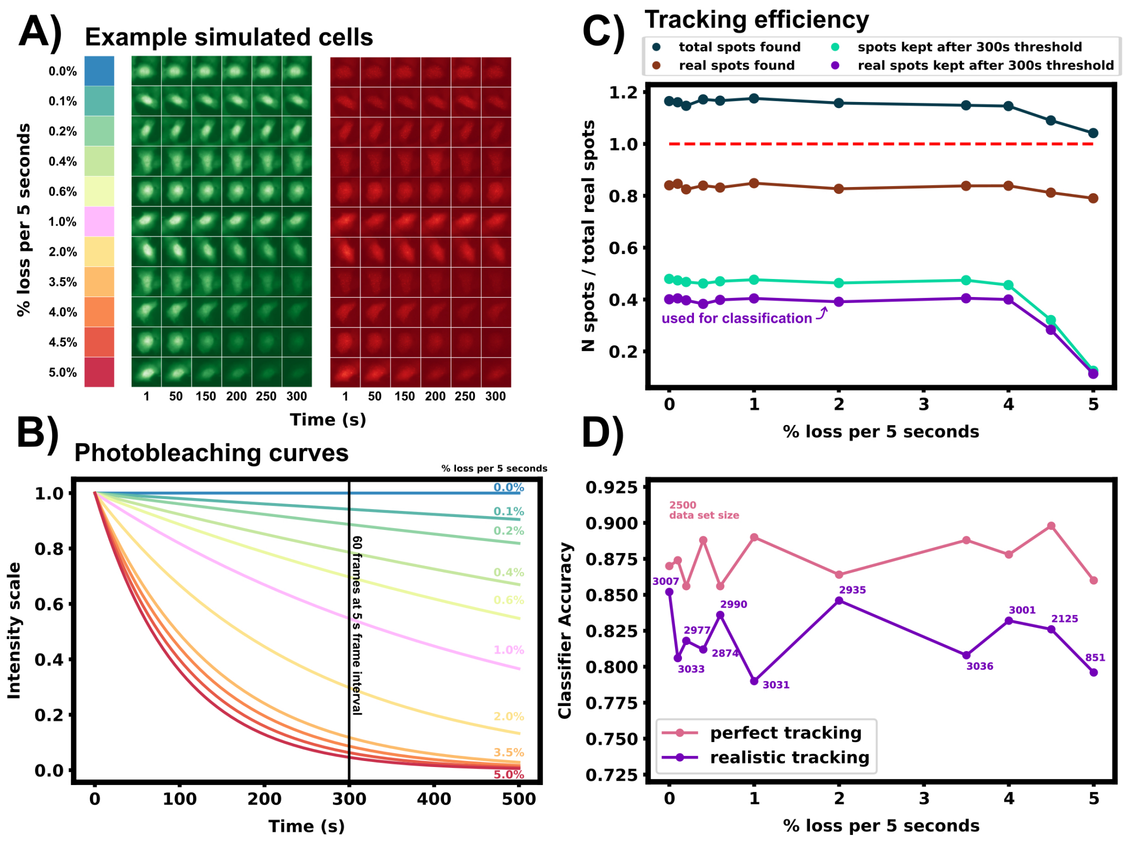

Figure S3. Effects of photobleaching and tracking on machine learning classification. A) Representative examples from 75 simulated cells of KDM5B and P300 at base conditions (Table 2) that were simulated at each of 11 different photobleaching rates. Tracking was performed on RNA spots in the red channel at a frame interval of 1 s. Translation was quantified in the green channel for 60 frames at a 5 s interval. B) Photobleaching intensity curves that scale video frames (red and green channels) to generate simulated cell videos (panel A). C) Efficiency for realistic tracking is reported as (# spots tracked / # total true spots). Total number of spots found by Trackpy per data set includes false positive spots, real trajectories, and real trajectories that are improperly linked or "fragmented," resulting in more spots being found than real simulated spots. When filtering with a squared error to real simulated spots, 80% of real spots are recovered in full or fragmented form (brown line). Requiring any spot, real or fake, to be tracked longer than 300 seconds results in ∼50% recovery until large photobleaching rates (green line). Filtering for spots with low matching error and existing longer than 300 seconds recovers ∼40% of true spots until larger photobleaching rates (purple line). The purple line represents the detected spot subset that was used for training in the "realistic tracking" condition. D) Classification accuracy versus photobleaching loss rate under perfect tracking (pink) or realistic tracking with image processing errors (purple). 2,500 spots were used for training in the perfect tracking case. For realistic tracking, the actual number of identified and true tracks varies and is shown in purple numbers for each photobleaching rate (out of 7,500 total generated spots). When training classifiers, 500 spots were withheld for testing classification accuracy.

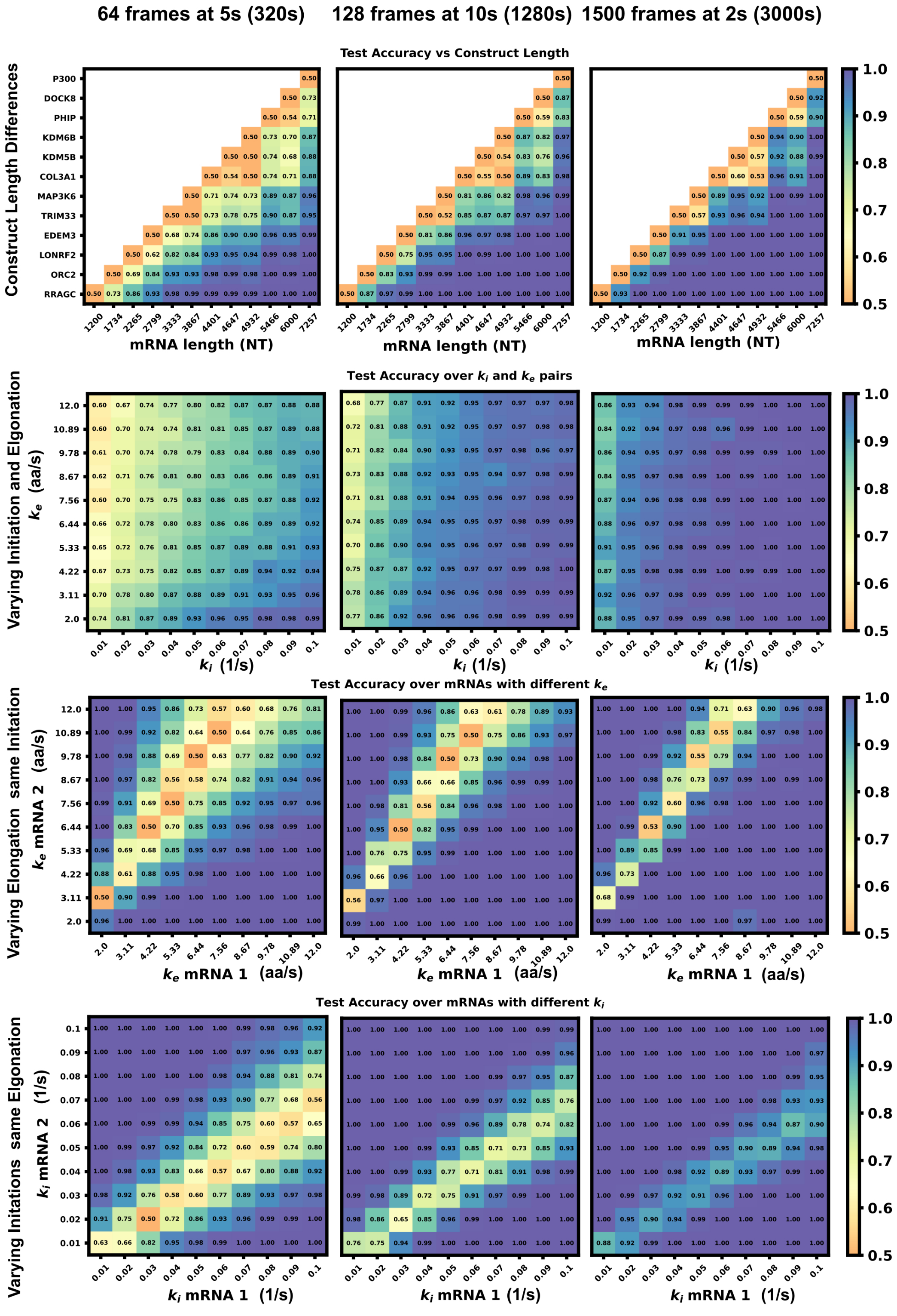

Figure S4. Machine learning accuracy for increased video size and resolution. Panels on left column are duplicated from Figure 5A-D in the main text (64 frames at 5s frame interval) and compared to the accuracy with increased video size of (middle) 128 frames at 10 s frame interval and (right) (1500 frames at 2 second frame interval)
